# Pharmacological targeting of bone marrow mesenchymal stromal/stem cells for the treatment of hematological disorders

**DOI:** 10.1186/s41232-017-0038-5

**Published:** 2017-04-03

**Authors:** Noriko Sugino, Tatsuo Ichinohe, Akifumi Takaori-Kondo, Taira Maekawa, Yasuo Miura

**Affiliations:** 1 Department of Transfusion Medicine and Cell Therapy, Kyoto University Hospital, 54 Kawaharacho, Shogoin, Sakyo-ku, Kyoto, 606-8507 Japan; 2 Department of Hematology/Oncology, Graduate School of Medicine, Kyoto University, Kyoto, 606-8507 Japan; 3 Department of Hematology and Oncology, Research Institute for Radiation Biology and Medicine, Hiroshima University, Hiroshima, 734-8553 Japan

**Keywords:** Mesenchymal stromal/stem cell, Hematopoiesis, Regeneration, Immunomodulation, Pharmacological modification, Cell therapy

## Abstract

The therapeutic effects of mesenchymal stromal/stem cells (MSCs) are mainly based on three characteristics: immunomodulation, tissue regeneration, and hematopoietic support. Cell therapy using culture-expanded MSCs is effective in some intractable bone and hemato-immune disorders; however, its efficacy is limited. In this article, we review the previous efforts to improve the clinical outcomes of cell therapy using MSCs for such disorders. We describe pharmacological targeting of endogenous bone marrow-derived MSCs as a crucial quality-based intervention to establish more effective MSC-based therapies.

## Background

There are two types of multipotent cells in bone marrow (BM): hematopoietic stem/progenitor cells (HSCs) and mesenchymal stromal/stem cells (MSCs). HSCs produce all types of hematopoietic cells and are established as a central player in BM. MSCs support hematopoiesis in the BM microenvironment and have been considered to be a second-class player in BM, despite their ability to differentiate into a variety of mesenchymal cells [1–4]. Nevertheless, emerging evidence has revealed the active contribution of BM-derived MSCs (BM-MSCs) to the pathogenesis of hematological diseases. More importantly, culture-expanded MSCs are practically available in clinics as off-the-shelf stem cell products for the treatment of some intractable refractory diseases. This review describes the basic characteristics of human MSCs and their clinical applications in the past and present and looks ahead toward the new horizon of MSC-based therapy.

## Main text

### Characteristics of human MSCs

**Fig. 1:**
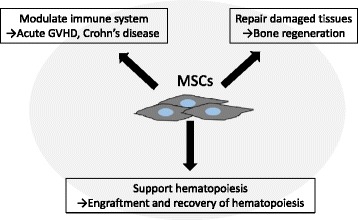
The main characteristics of MSCs. MSCs are multipotent stromal cells that have the ability to modulate the immune system, support hematopoiesis, and repair damaged tissues. These characteristics are applied to treat acute GVHD and Crohn’s disease, to regenerate bone, and to induce engraftment and recovery of hematopoiesis by infusing ex vivo expanded MSCs

The International Society of Cellular Therapy (ISCT) has proposed the following minimal criteria of human MSCs to define their characteristics [5]: (1) the ability to adhere to plastic plates; (2) the ability to differentiate into osteoblasts, adipocytes, and chondroblasts in vitro; and (3) the positive surface expression of CD105, CD73, and CD90 in the absence of surface human leukocyte antigen (HLA)-DR molecules and hematopoietic lineage markers of pan-leukocytes (CD45), endothelial/primitive cells (CD34), myeloid lineage cells (CD14 or CD11b), and B cell lineage cells (CD79α or CD19). MSCs are isolated from various tissues/organs via diverse methods in multiple institutions [6, 7]. Therefore, it is critical to determine the common characteristics of MSCs in order to discuss clinical and basic studies using these cells. The minimal criteria for MSCs proposed by the ISCT are appropriate for product identity but have no relevance to functions including hematopoietic support, immunomodulation, and tissue regeneration (Fig. 1).

There are two principal methods to isolate MSCs: classical isolation and prospective isolation. The classical isolation method selects cells that adhere to plastic dishes and form colonies. This method is simple and convenient; however, the isolated cells are heterogeneous. The prospective isolation method is based on cell sorting using surface markers that are expressed on MSCs [8, 9]. This method has the advantage of isolating a homogenous and high-quality cell population. According to the database provided by the National Institutes of Health (USA) at http://www.clinicaltrials.gov/, the conventional isolation method has been generally used in clinical trials using MSCs.

### Clinical applications of human MSCs

#### Acute graft-versus-host disease (GVHD)

A substantial proportion of patients who undergo allogeneic hematopoietic stem/progenitor cell transplantation (HSCT) develop intractable acute graft-versus-host disease (GVHD). The European Group for Blood and Marrow Transplantation conducted a multi-institutional phase II study and showed that infusion of MSCs from multiple donor sources conferred an overall response rate of 71% (39 of 55 cases), with a complete response rate of 55% and a partial response rate of 16%, in cases with steroid-resistant acute GVHD [10]. The 2-year overall survival rate in cases with a complete response was 52%, which was better than that in historical controls (about 10%). These results suggested that intravenous infusion of MSCs is an effective therapy for patients with steroid-resistant acute GVHD.

In clinical trials using commercial off-the-shelf MSC products, their infusion was tolerable overall and they showed an efficacy to improve acute GVHD, especially in pediatric patients and gastrointestinal GVHD patients [11–15]. However, the preliminary results of a phase III study that was conducted outside of Japan showed that infusion of MSCs had an initial effect, but conferred no significant advantage in the longer term for acute GVHD patients [16]. A recent meta-analysis of 13 studies (336 patients) revealed that 241 (72%) patients achieved an overall response, with a 6-month overall survival rate of 63% in responders versus 16% in non-responders [17]. The overall response rate of individual organs was 49% for the gastrointestinal tract, 49% for the skin, and 28% for the liver. Although MSCs are certainly effective for the treatment of acute GVHD, the results of long-term follow-up are needed.

#### Skeletal disorders

Osteogenesis imperfecta (OI) is an inherited skeletal dysplasia characterized by osteopenia and frequent bone fractures. The molecular mechanism underlying this disease is a defect of type I collagen (COL1a1 and COL1a2) in progenies of MSCs, namely, osteoblasts. Allogeneic BM transplantation effectively improved the histological and clinical manifestations of OI in children [18, 19]. However, the engraftment of donor cells was not ensured via this strategy. In 2005, Le Blanc et al. performed in utero transplantation (IUT) of MSCs into a female fetus with severe OI [20]. A bone biopsy after delivery showed the engraftment of donor cells, suggesting that IUT is a promising strategy to solve the problem of engraftment and settlement of donor-derived MSCs.

Hypophosphatasia (HPP) is an inherited metabolic disorder characterized by low alkaline phosphatase activity and impaired bone formation. BM transplantation transiently improved the clinical features of HPP, but a boost of donor BM cells was required [21]. Tadokoro et al. reported successful BM and MSC transplantation into an 8-month-old patient with perinatal HPP [22]. Subsequently, the same group reported that transplantation of ex vivo expanded allogeneic MSCs following BM transplantation improved bone mineralization, muscle mass, respiratory function, and mental development in patients with HPP [23]. Combined BM and MSC transplantation may be effective to prevent the rejection of allogeneic donor-derived MSCs.

Cell therapy using MSCs has been applied for bone regeneration in adults. One important application is the repair of bone fractures or defects due to malignant bone tumors or external injuries. Quatro et al. reported three cases of successful autologous BM stromal cell transplantation to treat large bone defects in the tibia, ulna, and humerus [24]. They expanded osteoprogenitor cells isolated from BM cells and implanted them into the lesion sites with macroporous hydroxyapatite scaffolds. All three patients achieved improvement of bone function and radiographic examination findings. Following this report, many studies of local MSC transplantation for bone repair were conducted. However, the osteogenic differentiation potential of implanted MSCs in defected lesions was not certified in these reports.

#### Hematopoietic engraftment and recovery after HSCT

Attempts have been made to use MSCs to support hematopoiesis upon HSCT. For this purpose, two major interventions were applied: co-transplantation of HSCs and MSCs and transplantation of HSCs that were expanded ex vivo in the presence of MSCs.

In an early phase I/II trial of co-transplantation of autologous peripheral blood stem/progenitor cells (PBSCs) and culture-expanded autologous MSCs in advanced breast cancer patients that received high-dose chemotherapy, engraftment was effectively accelerated [25]. Following this report, clinical trials of co-transplantation of allogeneic BM or PBSCs and MSCs for patients with hematological malignant diseases were conducted (Table 1) [26–28]. Lazarus et al. co-administered HSCs and culture-expanded MSCs from the same donor (HLA-identical siblings) after myeloablative conditioning; however, acceleration of engraftment was not observed [26]. Le Blanc et al. conducted a pilot study of co-transplantation of MSCs and HSCs for patients with graft failure [27]. All patients achieved engraftment, indicating that such co-transplantation improves engraftment of cells from the second donor in salvage HSCT. MacMillan et al. reported that co-transplantation of MSCs supported rapid engraftment of unrelated cord blood cells in children with high-risk leukemia [28]. In summary, although co-transplantation of MSCs is not effective in a standard risk transplantation setting, it could be effective in cases of engraftment failure or delayed hematopoietic recovery, such as HSCT from HLA-haploidentical donors, cord blood transplantation, and retransplantation.

**Table 1:** Clinical studies of co-infusion of MSCs with HSCs for hematopoietic recovery after hematopoietic stem/progenitor cell transplantation

Number of patients	Median age of patients, years (range)	HSC donor	MSC donor	MSC dose (×10^6^/kg)	Median time for Neut recovery (range)	Median time for Plt recovery (range)	Reference
46	44.5 (19–61)	HLA-matched sibling	HSC donor	1.0, 2.5, or 5.0	Neut >500/μl at day 14 (11–26)	Plt >20,000/μl at day 20 (15–36)	[26]
7	12 (1–44)	HLA-matched sibling in three cases Unrelated donor in three cases Cord blood in one case	HLA-matched sibling or HLA-haploidentical donor	1.0	Neut >500/μl at day 12 (10–28)	Plt >30,000/μl at day 12 (8–36)	[27]
8	7.5 (0.25–16)	Cord blood	HLA-haploidentical parent	0.9–5.0	Neut >500/μl at day 19 (9–28)	Plt >50,000/μl at day 53 (36–98)	[28]

*HLA* human leukocyte antigen, *HSC* hematopoietic stem/progenitor cell, *MSC* mesenchymal stromal/stem cell, *Neut* neutrophil, *Plt* platelet

MSCs support the expansion of cord blood cells in vitro [29]. de Lima et al. studied whether cord blood cells culture-expanded in the presence of MSCs effectively induce hematopoietic recovery upon double cord blood cell transplantation [30]. Cord blood cells from one unit with a smaller cell number were expanded in co-culture with MSCs. These manipulated cells were co-transplanted with non-manipulated cord blood cells from another unit with a larger cell number. The time-to-engraftment of neutrophils and platelets was shorter in these patients than in the historical controls, indicating that ex vivo expansion of cord blood cells with MSCs is an effective strategy to improve engraftment.

### Pharmacological targeting of endogenous BM-MSCs

In most clinical trials using allogeneic human MSCs, these cells were isolated from tissues/organs of volunteer donors, culture-expanded ex vivo, and intravenously infused into recipients. This intervention is a “quantity”-based approach to achieve therapeutic effects of MSCs. However, ex vivo expansion of MSCs might change their characteristics and reduce their quality. More importantly, a substantial proportion of intravenously infused donor MSCs become trapped within the lungs and are not distributed to the damaged tissues/organs of recipients [31]. There is obviously a limitation in the current strategy employed for cell therapy using MSCs because their effects are not dependent on the sustained settlement of infused cells or on proximate interactions with the target cells [32].

In a series of preclinical studies using model mice, we suggested that pharmacological treatment modifies the functions of endogenous BM-MSCs to achieve their therapeutic effects (Table 2) [33–37]. Acetylsalicylic acid (ASA), also known as aspirin, is a medication used to treat pain, fever, and inflammation. These therapeutic effects are mediated through inhibition or modification of cyclooxygenases [38, 39]. We showed that treatment with ASA ameliorates bone loss in osteoporotic mice due to the increased bone-forming capability of ASA-treated BM-MSCs [33]. Telomerase activity is enhanced in ASA-treated BM-MSCs [33]. This observation is consistent with a previous report that ASA contributes to the improvement of bone mineral density, although the contribution of MSCs is unknown [40]. These preclinical and clinical studies indicate the efficacy of ASA treatment for bone repair in patients with skeletal disorders through activation of endogenous BM-MSCs.

**Table 2:** The effects of pharmacological treatment of MSCs

Drug	Target cells	Clinical effect	MSC-mediated hematopoiesis	MSC-mediated bone regeneration	Mechanism(s) in MSCs	References
ASA	Broad cells	Anti-inflammation	N/T	↑	Telomerase activity↑	[33]
EPO	Erythroid progenitors	Erythropoiesis	↑	↑	EPOR/Stat5 pathway↑	[34]
PTH	Osteoblasts/Osteoclasts	Osteoporosis	↑	→	CDH11 expression↑	[35]
VK2	Osteoblasts	Osteoporosis	↑	↑	CXCL12 expression↓	[37]
OICS	N/A	Osteoporosis	↑	→	CXCL12 and VCAM1 expression↓	[36]

Up arrows indicate up-regulation or activation. Down arrows indicate down-regulation or inactivation

*ASA* acetylsalicylic acid (aspirin), *EPO* erythropoietin, *EPOR* erythropoietin receptor, *MSC* mesenchymal stromal/stem cell, *N/T* not tested, *OICS* osteo-inductive cocktail (dexamethasone, phosphate, and vitamin C ), *PTH* parathyroid hormone, *VCAM1* vascular cell adhesion protein 1, *VK2* vitamin K2

Parathyroid hormone (PTH) is clinically used to treat osteoporosis because it has anabolic effects on bone formation though activating osteoblasts [41]. We demonstrated that short-term administration of PTH prolongs the survival of lethally irradiated mice that undergo BM transplantation, which is accompanied by enhanced hematopoietic marrow formation in BM [35]. PTH acts on human BM-MSCs to enhance their hematopoietic cell expansion capability through upregulation of the adhesion molecule cadherin-11 in BM-MSCs [35]. In another study, we showed that an erythropoiesis-stimulating agent, erythropoietin, acts on human BM-MSCs to enhance not only bone formation but also hematopoietic marrow formation in vivo, by using ectopically xeno-grafted mice [34]. The erythropoietin receptor/Stat5 pathway is enhanced in BM-MSCs as well as in erythroblast progenitor cells [34, 42]. Vitamin K2 (VK2) is clinically approved for the treatment of patients with osteoporosis. It is known that VK2 improves hematopoiesis in some patients with hematological diseases although the underlining mechanisms are not fully understood [43, 44]. In our study, the expression of CXCL12 in VK2-treated BM-MSCs was low, which suggested that CXCL12-CXCR4-mediated interaction between BM-MSCs and HSCs is released, thereby HSCs expand and differentiate into mature hematopoietic cells [37].

**Fig. 2:**
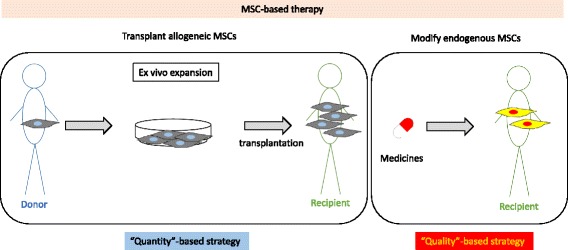
MSC-based therapy with pharmacological modification of endogenous MSCs. In a conventional approach, MSCs are isolated from donors, culture-expanded ex vivo, and then infused into recipients, mainly intravenously. This intervention is a “quantity”-based strategy to achieve the therapeutic effects of MSCs (*left panel*). We have proposed a strategy in which pharmacological treatment activates or modifies the functions of endogenous MSCs. This intervention is a “quality”-based strategy to achieve the therapeutic effects of MSCs (*right panel*)

We have proposed that pharmacological targeting of endogenous MSCs is a quality-based intervention to achieve therapeutic effects in patients (Fig. 2). This strategy may enhance the therapeutic capability of MSCs to act closely on target cells through secretion of soluble factors and adherence in microenvironments, without requiring the redistribution of externally infused MSCs to damaged tissues/organs. However, attention needs to be paid to unexpected off-target effects of drugs in patients. To avoid this, we have sought drugs that act on MSCs and elicit therapeutic effects among compounds developed for medical purposes. We believe that this drug repositioning strategy shortens the drug development period, reduces medical costs, and provides patients with safe medications. In addition, there is a possibility that the characteristics of MSCs in patients might be affected [45]. Therefore, pharmacological stimulation of such affected MSCs may have unexpected effects on the pathogenesis of diseases. Thus, further investigations are needed to establish a quality-based, pharmacological, MSC-targeted strategy.

### Perspectives of MSC-based therapy

We recently reported that short-term treatment with ascorbic acid, inorganic phosphate, and dexamethasone (osteogenesis-inducing cocktails) accelerates hematopoietic recovery in mice that undergo BM transplantation, with altered chemotaxis- and adhesion-related gene expression profiles in BM-MSCs [36]. As well as treatment with a single pharmacological agent, combination treatment is also effective to achieve a therapeutic effect.

Recent studies reveal that MSCs are associated not only with normal hematopoiesis but also with the pathogenesis and progression of hematological malignant diseases. Our laboratory previously reported that defective MSCs are responsible for the impaired physiological early B cell lymphopoiesis in C/EBPβ-knockout mice [46]. Furthermore, MSC-mediated resistance to anti-cancer drugs in B cell precursor acute lymphoblastic leukemia cells can be ameliorated by pharmacological treatment of MSCs [47]. Raaijmakers et al. showed that deletion of *Dicer1* in mouse osteoprogenitors causes myelodysplasia [48]. Balderman et al. suggested a novel therapeutic strategy to target the BM microenvironment for the treatment of myelodysplastic syndromes using model mice [49]. Collectively, the BM microenvironment is closely related to the pathogenesis and progression of hematological malignant diseases; therefore, targeting MSCs in this microenvironment is a crucial therapeutic strategy.

## Conclusions

MSCs have a variety of biological characteristics. Cell therapy using MSCs is effective in a substantial proportion of intractable diseases; however, it is still in the process of development. Further investigations are needed to establish more effective MSC-based therapies.

## Abbreviations

ASA: Acetylsalicylic acid
BM: Bone marrow
BM-MSC: Bone marrow-derived mesenchymal stromal/stem cell
GVHD: Graft-versus-host disease
HLA: Human leukocyte antigen
HPP: Hypophosphatasia
HSC: Hematopoietic stem/progenitor cell
HSCT: Hematopoietic stem/progenitor cell transplantation
ISCT: International Society of Cellular Therapy
IUT: In utero transplantation
MSC: Mesenchymal stromal/stem cell
OI: Osteogenesis imperfecta
PBSC: Peripheral blood stem/progenitor cells
PTH: Parathyroid hormone
